# Pedestrian smartphone overuse and inattentional blindness: an observational study in Taipei, Taiwan

**DOI:** 10.1186/s12889-018-6163-5

**Published:** 2018-12-31

**Authors:** Ping-Ling Chen, Chih-Wei Pai

**Affiliations:** 1 Graduate Institute of Injury Prevention and Control, College of Public Health, Taipei Medical University, Taipei, Taiwan; 2 Graduate Institute of Injury Prevention and Control, College of Public Health, Taipei Medical University, 250 Wu-Shing Street, Taipei, Taiwan

**Keywords:** Smartphone gaming, Pedestrian safety, Inattentional blindness, Smartphone overuse

## Abstract

**Background:**

Smartphone addiction has become a crucial social issue. Past studies have indicated that phone use such as talking or texting while walking constitutes a dual task that may cause pedestrians inattentional blindness and impair their awareness of surroundings.

**Methods:**

This study investigated the influence of various smartphone tasks (calling, music listening, texting, playing games, and web surfing) on the smartphone overuse and inattentional blindness of pedestrians in Taipei, Taiwan. Pedestrian smartphone overuse was observed and recorded via WiFi cameras to determine whether pedestrians were using their smartphones when crossing a street with a signal. After crossing the street, pedestrians were interviewed to obtain additional information regarding demographics, smartphone tasks, data plan, and screen size. Pedestrians were classified into the case (distracted) and control (undistracted) groups. By determining whether pedestrians saw something unusual—a clown walking the opposite direction—and heard the national anthem played by the clown, inattentional blindness and deafness were examined. Pedestrians’ situational awareness was assessed by ascertaining whether they remembered how many seconds remained before the crossing signal upon arriving at the curb.

**Results:**

In total, 2556 pedestrians crossed the street and underwent the interview. Smartphone overuse and inattentional deafness were the commonest among music listeners. Playing Pokémon Go gaming was the task most associated with inattentional blindness. Logistic regression models revealed that contributing factors to smartphone overuse and inattentional blindness were a large smartphone screen (≥5 in), unlimited mobile Internet data, and being a student. The interactions of gaming with being a student and with unlimited data were significantly associated with smartphone overuse, inattentional blindness and deafness, and situational awareness.

**Conclusions:**

Listening to music was the smartphone task most associated with pedestrian smartphone overuse and inattentional deafness. Pokémon Go was the most associated task with inattentional blindness and reduced situational awareness.

## Background

Smartphone addiction can be considered the uncontrollability of smartphone use despite significant harmful financial, physical, psychological, and social consequences [1]. In recent years, smartphone addiction, particularly among adolescents, has become a crucial social issue [2–4]. Adolescents, compared with adults, were reported to be at greater risks of the undesirable consequences because adolescents are yet to develop self-control in smartphone use [2].

Numerous studies have been conducted examining the factors influencing smartphone addiction. Conducting an online questionnaire survey of students from a vocational high school in Taiwan, Liu et al. [3] revealed that students using smartphones for predominantly gaming or for gaming and multiple other applications were the most susceptible to smartphone addiction. Employing a face-to-face interview survey of middle-school students in Korea, Cha and Seo [2] reported that using mobile messengers is the most influential determinant of smartphone addiction, followed by web surfing, gaming, and social networking. From a questionnaire survey of Swiss vocational schools, Severin et al. [4] reported that social networking was the most relevant smartphone function associated with smartphone addiction. Kim et al. [5] conducted an online survey of 608 college students in Korea and estimated their smartphone addiction with a standardized measure realized by Korea’s National Information Society Agency, namely the Smartphone Addiction Proneness Scale. Kim et al. reported that people with smartphone addiction were more likely to experience an accident, fall from height, or bump than those without addiction.

Various smartphone tasks including web surfing, music listening, gaming, and texting are frequent smartphone activities associated with lower situational awareness as well as higher cognitive distraction, occasionally endangering the lives of users [6]. For instance, past studies [7–9] have suggested that texting interferes significantly more with walking than does reading news, talking, or listening to music on a smartphone. Pedestrians texting, compared with those talking or listening to music, could not maintain their walking pace as closely and were more likely to amble across the street [10]. Byington and Schwebel [9] conducted a laboratory experiment and reported that compared with standard pedestrians, those surfing the Internet missed more opportunities to cross roads safely, waited longer before crossing, looked away from the street more, looked both ways less frequently, and were more prone to experience or nearly experience a collision with a vehicle in a virtual environment.

A situational awareness study related to various smartphone tasks was conducted by Haga et al. [11], who tested 24 university students texting, playing simple games, and watching videos. They examined students performing simultaneous auditory and visual detection tasks. Those playing games on their smartphones missed the most visual targets and exhibited the worst ability to walk and balance [11]. Similar findings were reported by Hyong [12], who determined that cognitive ability was significantly reduced when gaming on phones, which led to the largest reduction in dynamic balance, followed by texting, web surfing, and music listening. A similar laboratory study was employed by Lin and Huang [8], who concluded that while walking, reading on an app reduced situational awareness and increased perceived workload more than a picture-dragging task. De Waard [13] evaluated the detrimental effects of simultaneous smartphone gaming and biking on a public cycling path and found a correlation with swerving.

Chen et al. [14] executed a real-life observational study to examine the effects of various smartphone tasks (e.g., talking, texting, web surfing, and gaming) on pedestrian behavior and reported that playing Pokémon Go exhibited the strongest association with several risky street-crossing behaviors, such as not using the designated crossing or crossing on red. More recently, several types of smartphone game (i.e., action, racing, shooters, sports, and Pokémon Go) were examined specifically by Chen and Pai [15], who concluded that Pokémon Go and racing games were the first and second most associated with pedestrians crossing on red or outside the designated pedestrian crossing, respectively.

Inattentional blindness resulting from phone use while walking has also received extensive attention in psychology literature. Hyman et al. [16] found that walking participants did not notice a clown on a unicycle. Hyman et al. [17] subsequently found that when simultaneously texting and walking, participants were less likely to notice an unusual object (money in a tree) near the pathway. This inattentional blindness was attributed to decreased awareness resulting from a division of focus in a complicated environment; exciting and unusual objects outside a person’s focus may go unnoticed because they are unrelated to a main task.

Research has implied that smartphone addiction has become a social issue, particularly among adolescents. In addition, various smartphone tasks such as texting while walking were identified to induce inattentional blindness and reduce situational awareness. Smartphone technology has rapidly advanced—including larger screen sizes that are beneficial for streaming video or gaming and the fourth generation (4G) of telecommunications technology—which allows for faster transmission of data [18]. Consequently, excessive smartphone use when walking can be more cognitively demanding than it was before, causing inattentional blindness and decreased situational awareness to a greater degree.

### Purpose

This study evaluated the influence of various smartphone activities (including gaming, music listening, talking, texting, and web surfing) on the smartphone overuse, inattentional blindness, and situational awareness of pedestrians.

## Methods

### Participants and procedures

We adopted the same data collection method used by Chen and her colleagues [14, 15], who have both observed and surveyed their participants seen crossing the street while using their smartphones. The data collection process is described as follows.

The first step in data collection was to observe pedestrians’ smartphone overuse when crossing the street. On each side of a road, two camera devices (relevant model: D-Link DCS-2630 L Full HD 180-Degree Wi-Fi Camera) were installed to record video. To avoid being spotted by the pedestrians, the video cameras were well hidden. Pedestrians who were crossing the street and were observed to be using their phones were classified into the case group (distracted group), and those not using phones were in the control group (undistracted group). Smartphone overuse was defined as using the smartphone while crossing the street. We only included those presented with a red light.

The second step in data collection was to interview pedestrians who had crossed the street. A random approach to pedestrian selection is crucial to avoid bias. Pedestrians were chosen at random by an Internet random number generator. After observers watched the video in real time and performed the sampling, the interviewers were told which pedestrians to invite for interview. Both undistracted and distracted pedestrians were selected through this process. Immediately after crossing the street, pedestrians were interviewed about their smartphone task, screen size, data plan, and demographic information. The tasks examined included listening to music, traditional phone calling, using social media apps (e.g., Facebook, YouTube, Instagram), using messaging apps (texting, voice talking, video talking), web surfing (reading news/emails, checking a map), and smartphone gaming. If multiple pedestrians were found using their devices, one was selected at random for interview.

In addition to smartphone overuse, the inattentional blindness and situational awareness of pedestrians were evaluated. We hypothesized that different smartphone tasks are associated with inattentional blindness and deafness to a different degree. A research assistant was recruited to wear a clown outfit and walk in the opposite direction of the pedestrians while playing the Taiwan national anthem at approximately 60 dBA (at a distance of 1 m) from a smartphone carried in one hand. The clown served as an unusual stimulus to evaluate pedestrians’ inattentional blindness, and the national anthem was for examining whether smartphone tasks are associated with inattentional deafness. We also hypothesized that during dual-task walking, pedestrians’ situational awareness is impaired by smartphone tasks. In our study, situational awareness was evaluated by investigating whether pedestrians forgot the number of seconds remaining for the red signal upon arriving at the curb. Pedestrians who completed crossing the street were interviewed to learn if they had seen the clown, heard the national anthem, or remembered the remaining seconds on the red signal. Notably, the case group (i.e., distracted group) also comprised those who finished their smartphone use prior to crossing the street because cognitive distraction may still have occurred.

Those jogging across the street and those using smartphones with keyboards were excluded from our study. Pedestrians aged less than 18 were removed from the study. As an incentive, all interviewees were offered a gift for their participation, such as a pen or a notebook (price: ~US$1). The Institutional Review Board that is affiliated with Taipei Medical University ratified our study.

**Fig. 1:**
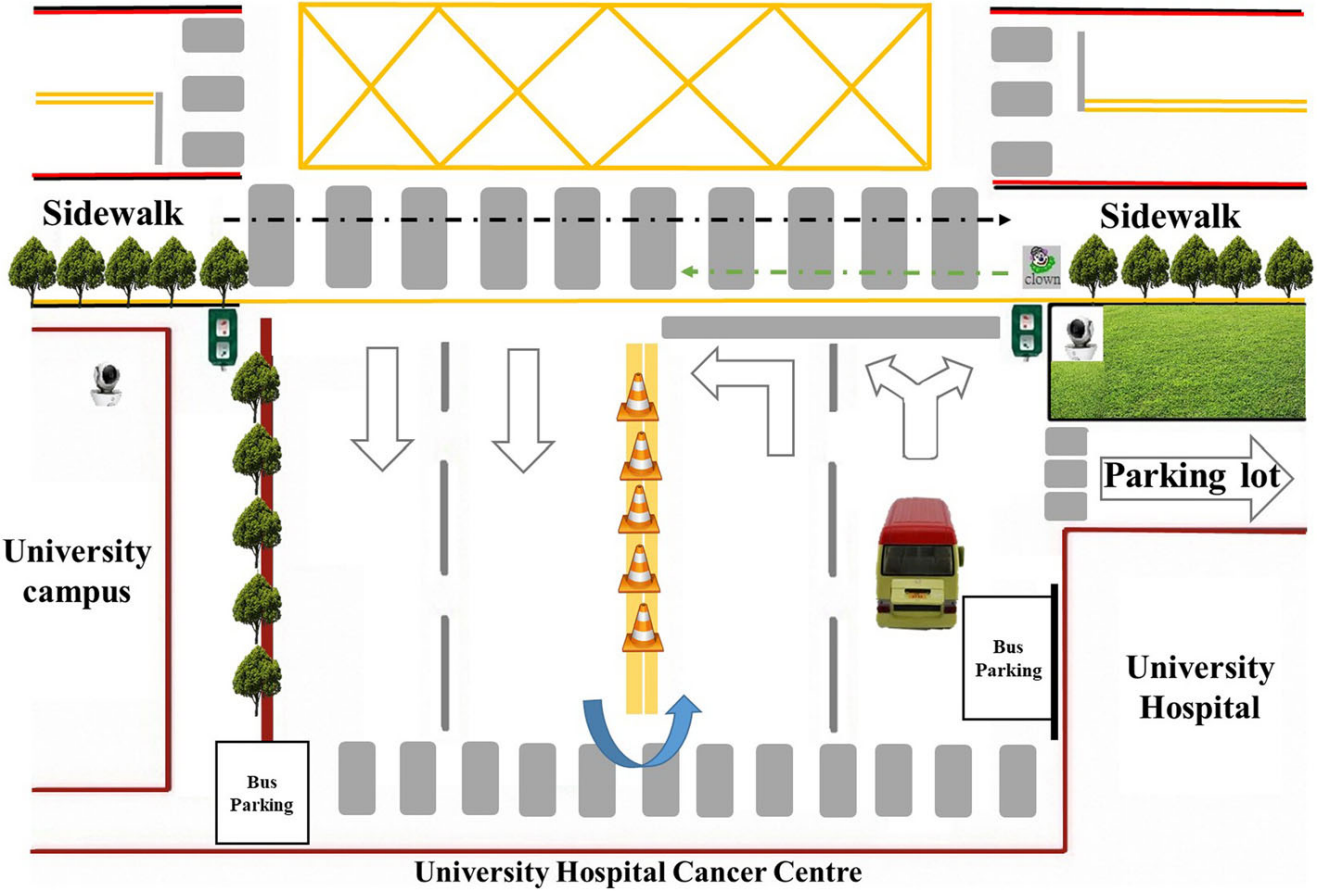
Intersection that served as the location for pedestrian observations and interviews*.* This figure has been reproduced from a figure in our past publications [14, 15, 27] with permissions obtained from the publishers

Data were collected from August 2016 to July 2017, during which three periods of day were considered: early rush hour (07:00–09:00), nonpeak hours (12:00–14:00), and finally evening rush hour (16:00–18:00). The university provided consent to use three intersections; however, because of money and manpower limitations, only one intersection was chosen at random, and this intersection connected the university hospital to the university. The selected 18-m intersection (Fig. 1) was controlled by automatic pedestrian signals on 90-s loops, with 25 s on green and 65 s on red; a countdown signal device showed the remaining seconds. The speed limit on the intersecting streets was 20 km/h.

### Variables considered

Student status, monthly data limit, age, smartphone task, smartphone screen size, and gender constituted the measured independent variables. Among other occupations, the pedestrians included university staff, doctors, students, and hospital or university administrators. The literature suggests that students are more likely than others to exhibit smartphone overuse [9]; therefore, pedestrians were classified as either students or others for the “student status” variable.

We considered only one continuous variable, which was age (y). We also examined a temporal variable, which was the time of observation: off-peak hours (09:01–16:59) or rush hours (07:00–09:00 and 17:00–19:00). As a categorical variable, screen size was divided into screens 5 in. (diagonal length) or larger and screens smaller than 5 in.. Mobile Internet data limit was classified as none, restricted, or unlimited. Age data as means and standard deviations are presented in Table 1.

**Table 1:** Proportions of smartphone tasks and associated independent variables (*N* = 2556)

Characteristics	Smartphone tasks									Total (%)
	Control (%)	Listening to music (%)	Talking (traditional) (%)	Using social media (%)	Video talking via an app (%)	Voice talking via an app (%)	Texting (using an app) (%)	Web surfing (%)	Gaming (%)	
Gender										
Male	179(52.5)	74(51.8)	96(47.3)	48(42.1)	35(41.7)	147(47.6)	189(45.9)	70(61.4)	438(52.5)	1276(49.9)
Female	162(47.5)	69(48.3)	107(52.7)	66(57.9)	51(59.3)	162(52.4)	223(54.1)	44(38.6)	396(47.5)	1280(50.1)
Occupations										
Students	157(46.0)	121(84.6)	127(62.6)	71(62.3)	58(67.4)	218(70.6)	257(62.4)	75(65.8)	527(63.2)	1611(63.0)
Others	184(54.0)	22(15.4)	76(37.4)	43(37.7)	28(32.6)	91(29.4)	155(37.6)	39(34.2)	307(36.8)	945(37.0)
Age (in years) ^a^	m: 31.8; s: 12.1	m: 20.3; s: 7.9	m: 29.8; s: 13.6	m: 25.5; s: 8.4	m: 24.1; s: 7.2	m: 21.2; s: 6.7	m: 24.2; s: 6.4	m: 20.3; s: 5.2	m: 23.6; s: 9.0	m: 25.1; s: 7.7
Screen size										
< 5 in.	114(33.4)	41(28.7)	106(52.2)	46(40.4)	21(24.4)	139(45.0)	174(42.2)	51(44.7)	371(44.5)	1063(41.6)
> =5 in.	227(66.6)	102(71.3)	97(47.8)	68(59.6)	65(75.6)	170(55.0)	238(57.8)	63(55.3)	463(55.5)	1493(58.4)
4G Internet data allowance										
Unlimited use	202(59.2)	76(53.1)	110(54.2)	57(50.0)	46(53.5)	179(57.9)	267(64.8)	60(52.6)	644(77.2)	1641(64.2)
Restricted use	103(30.2)	31(21.7)	52(25.6)	39(34.2)	24(27.9)	83(26.9)	104(25.2)	33(28.9)	182(21.8)	651(25.5)
None	36(10.6)	36(25.2)	41(20.2)	18(15.8)	16(18.6)	47(15.2)	41(10.0)	21(18.4)	8(1.0)	264(10.3)
Total (%)	341(13.3)	143(5.6)	203(7.9)	114(4.5)	86(3.4)	309(12.1)	412(16.1)	114(4.5)	834(32.6)	2556

^a^*m* = mean, *s* = standard deviation

This study investigated several smartphone tasks including listening to music, texting (including messaging apps), voice calls (including voice call apps), video calls, using social media apps, web surfing, and gaming (any smartphone game). Traditional texting was later excluded from the analysis because only three pedestrians reported that they were texting via standard telecommunication networks. A vast majority of participants reported playing Pokémon Go; therefore, other non–augmented reality (AR) games, as well as other games such as Candy Crush, were removed from the analysis because too few (*n* = 24) pedestrians reported playing them to yield statistical significance in our regression models.

### Analysis

We first analyzed the distribution of the various smartphone tasks. Subsequently, smartphone overuse, inattentional blindness and deafness, and situational awareness were cross tabulated with the smartphone tasks. The percentages of other outcome variables among the tasks were subjected to chi-square testing post hoc to determine significant differences. Next, using logistic regression models, we examined the factors predicting the outcome variables: smartphone overuse, inattentional blindness and deafness, and situational awareness. All multivariate regression analyses were conducted using logistic regression (for the binary variables, such as smartphone overuse).

First, using Internet data limit, screen size, gender, occupation, and age, we performed univariate regressions. For multivariate regression, we included all significant (*p* < 0.2) variables from the univariate regressions. For conciseness, univariate regressions results are not presented; significant variables were retained for the final regression analyses.

## Results

### General results

**Fig. 2:**
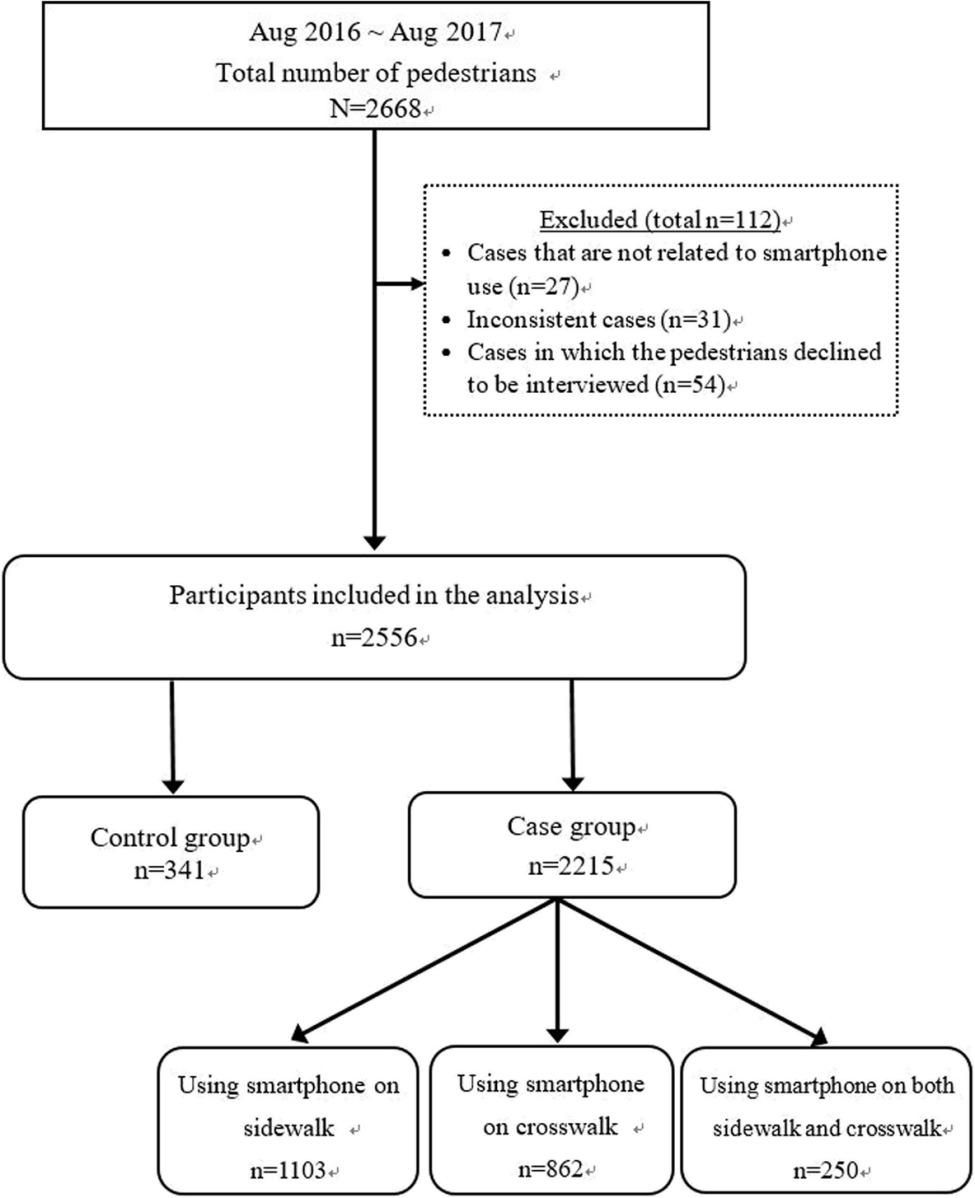
Flowchart

Figure 2 illustrates the flowchart of sampling. Overall, we observed 2668 pedestrians. We excluded 27 cases of pedestrians using other electronic gadgets, such as smart watches, compact radios or MP3 players, or smartphones with keypads. We further excluded 69 cases of participants seen using their smartphones who claimed not to. In 54 cases, the participants refused to be interviewed; these were also excluded. Finally, 2556 cases were considered valid and included; the created control group comprised 341 pedestrians, and the created case group comprised 2215 pedestrians. Of the 2215 participants, 1103 were using smartphones on the sidewalk while waiting for the light but stopped when they began to cross the street, 862 used their phones while crossing the street, and 250 used their smartphones nonstop both while waiting and crossing.

The smartphone tasks and associated independent variables are listed in Table 1. Of the participants, 32.6% claimed to be playing Pokémon Go, and 16.1 and 12.1% were texting and voice calling via an app, respectively. Interestingly, of those gaming on their phones, 77.2% had no Internet data limit (the highest proportion found among all smartphone tasks), 63.2% were students, and 55.5% had larger screens.

The proportions of smartphone overuse, inattentional blindness and deafness, and situational awareness by various smartphone tasks are presented in Table 2. Notably, the control group for overuse (i.e., using while crossing) was those using their phones for traditional talking, not an undistracted group. The control group was not distracted for the other three outcome behaviors: not seeing the clown, not hearing the song, and forgetting the remaining seconds. The numbers for the control for these three behaviors would never be zero, because we interviewed these undistracted pedestrians to learn whether they saw the clown, heard the song, or remembered the remaining seconds. Smartphone overuse (i.e., using smartphone when crossing) was exhibited by 92.5% of music listeners, followed by 72.5% of Pokémon Go players. Smartphone game players were least likely to see the clown (45.7%; *p* < 0.01) or remember the remaining seconds (66.7%; *p* < 0.01), and music listeners least often heard the song (65.7%; *p* < 0.01).

**Table 2:** Outcome behaviors and different distracting activities (*N* = 2556)

	Using smartphone when crossing (%) (*n* = 1112)#	Failure to see the clown (%)(*n* = 631)	Failure to hear the song (%)(*n* = 766)	Failure to remember the remaining seconds (%)(*n* = 823)
Control	–	2.9	4.2	6.2
Listening to music	92.5^2^	5.9*	65.7**	8.4
Talking (traditional)	9.6	8.9*	16.5*	9.2*
Using social media apps	16.8^1^	12.1*	19.8	30.1*
Video talking via an app	12.4	35.6*	16.7**	27.6*
Voice talking using an app	40.4^1^	16.5	16.4**	10.6*
Text messaging using an app	43.8^2^	25.1**	12.5**	26.2**
Web surfing	18.2^1^	21.7*	16.8*	32.7*
Gaming	72.5^1^	45.7**	57.6**	66.7*

*p < 0.05 in relation to created control group

**p < 0.01 in relation to created control group

# Control group not included; ^1^ *p* < 0.05 in relation to talking (traditional); ^2^ *p* < 0.01 in relation to talking (traditional)

### Smartphone overuse and inattentional blindness

Table 3 reports the frequency of smartphone overuse, inattentional blindness, and situational awareness. Pokémon Go was the task most likely to result in failure to see the clown (odds ratio [OR] = 2.46, *p* < 0.01) and failure to remember the remaining seconds (OR = 2.33, *p* < 0.01). Participants listening to music were those who most frequently exhibited smartphone overuse (OR = 3.22, *p* < 0.01) and failed to hear the song (OR = 2.94, *p* < 0.01). After music listeners, Pokémon Go players were also those most commonly engaged in smartphone overuse (OR = 2.81, *p* < 0.01) and failing to hear the song (OR = 2.31, *p* < 0.01).

**Table 3:** Odds of using smartphone when crossing, inattentional blindness, inattentional deafness, and situational awareness (*N* = 2556)

	Using smartphone when crossing	Failure to see the clown		Failure to hear the horn		Failure to remember remaining secs	
	OR(95% CI) *P*-value	OR(95% CI)	*P*-value	OR(95% CI)	*P*-value	OR(95% CI)	*P*-value
Smartphone tasks (ref. control)							
Listening to music	3.22(2.57, 4.13) < 0.01	1.13(0.84, 1.26)	0.21	2.94(2.34, 3.28)	< 0.01	1.26(1.11, 1.38)	0.02
Voice talking (using an app)	1.12(0.87, 1.31) 0.17	1.22(1.06, 1.45)	0.03	1.86(1.55, 2.16)	< 0.01	1.21(0.80, 1.42)	0.21
Talking (traditional)	Ref.	1.18(0.94, 1.32)	0.16	1.95(1.79, 2.32)	< 0.01	1.30(0.74, 1.39)	0.33
Using social media apps	1.39(1.12, 1.78) 0.02	1.27(1.11, 1.54)	< 0.01	1.35(0.94, 1.48)	0.25	1.79(1.54, 2.21)	< 0.01
Video calling (using an app)	0.82(0.63, 1.08) 0.22	2.31(1.99, 2.67)	< 0.01	1.90(1.70, 2.25)	< 0.01	1.52(0.94, 1.68)	0.28
Texting messages (using an app)	1.57(1.19, 1.87) < 0.01	1.76(1.50, 2.01)	< 0.01	1.49(1.20, 1.85)	< 0.01	1.70(1.54, 2.16)	< 0.01
Web surfing	1.12(0.74, 1.36) 0.19	1.68(1.54, 1.90)	0.02	1.39(0.84, 1.46)	0.20	1.39(0.84, 1.46)	0.20
Gaming	2.81(2.24, 3.37) < 0.01	2.46(2.10, 2.86)	< 0.01	2.31(1.80, 2.74)	< 0.01	2.33(1.75, 2.86)	< 0.01
Students (ref. otherwise)	1.85(1.29, 2.24) < 0.01	1.27(1.08, 1.64)	< 0.01	1.26(0.89, 1.36)	0.16	1.39(1.16, 1.94)	< 0.01
Students x gaming (ref. otherwise)	2.21(2.01, 3.15) < 0.01	1.94(1.64, 2.31)	< 0.01	2.19(1.84, 2.39)	< 0.01	1.87(1.50, 2.24)	< 0.01
Screen size of 5 in. or larger (ref. otherwise)	1.67(1.26, 2.52) < 0.01	2.05(1.84, 2.52)	< 0.01	1.55(1.21, 1.68)	0.03	1.79(1.36, 2.27)	< 0.01
4G Internet data allowance (ref. none)							
Unlimited use	2.60(1.94, 3.37) < 0.01	1.86(1.51, 2.09)	< 0.01	2.08(1.84, 2.45)	< 0.01	1.53(1.22, 1.95)	< 0.01
Restricted allowance	1.23(0.90, 1.39) 0.24	1.22(0.94, 1.40)	0.23	1.16(0.88, 1.35)	0.33	1.06(0.78, 1.29)	0.28
Unlimited data x gaming (Ref. otherwise)	2.54(0.03, 3.23) < 0.01	2.33(1.95, 2.69)	< 0.01	1.60(1.31, 1.98)	< 0.01	2.16(1.84, 2.57)	< 0.01
*ρ* ^2^	0.34	0.31		0.29		0.27	

An interaction between smartphone gaming and student status influenced smartphone overuse, inattentional blindness and deafness, and situational awareness; student smartphone gamers had a 121% higher likelihood of using their phones when crossing the street, had a 94% higher likelihood of not seeing the clown, had a 119% higher likelihood of not hearing the clown, and had an 87% higher likelihood of forgetting the remaining seconds.

Those with unlimited Internet engaged in smartphone overuse, suffered from inattentional blindness and deafness, and exhibited impaired situational awareness respectively 2.6, 1.86, 2.08, and 1.53 times as more likely as participants without mobile Internet. Participants with 5-in. or larger screens demonstrated more smartphone overuse (OR = 1.67), inattentional blindness and deafness (ORs = 2.05/1.55), and decreased situational awareness (OR = 1.79) than did those with smaller screens. We observed a significant interaction of gaming and unlimited data; smartphone players possessing unlimited Internet data allowance were determined to be 2.54 times more likely to use their smartphones when crossing. This interaction term was also found to contribute to pedestrians’ visual and auditory inattention (ORs = 2.33/1.60) and decreased situational awareness (OR = 2.16).

## Discussion

Past studies [3, 19, 20] have established that social network apps, smartphone gaming, and adolescence are risk factors for pathological and compulsive smartphone use. We extend the understanding of traffic safety by concluding that listening to music and playing Pokémon Go are the smartphone activities that are most and second most associated with smartphone overuse, respectively. Chen and Pai [15] similarly reported that among several types of smartphone game, Pokémon Go was most likely to induce smartphone overuse. Although the measure of smartphone use while crossing the street was adopted as a surrogate indicator of smartphone addiction, it constitutes a risky behavior that may result in traffic crash and should not be overlooked when considering interventions for injury prevention. One likely reason for the effect of listening to music is that music listeners are likely to underestimate the likelihood of such behavior to cause a crash. This is corroborated by research [21] that reported that participants distracted by music were more frequently struck by automobiles in a virtual pedestrian environment than were other undistracted participants.

To supplement studies that have generally examined Internet or smartphone addiction or overuse among adolescents, we conclude that university students, who are older than adolescents, also appear to have smartphone overuse tendencies. Our findings suggest the need for intervention studies to monitor several certain groups of users, such as students, smartphone gamers, and smartphone music listeners, especially when crossing a street. Moreover, there is an urgent need for music listeners to be aware of the increasing number of quiet electric vehicles [22] that may constitute a silent hazard.

Psychological studies [16, 23] have established that phone use is associated with lower awareness of surroundings and inattentional blindness. Additionally, researchers in behavioral science, such as Danielle et al. [24] and Chen and Pai [15], have found that an increase in smartphone game complexity is associated with risk-taking street-crossing behaviors, such as accepting a narrower traffic gap and crossing during red lights. Advancing these studies, our current research successfully identified playing Pokémon Go as the task most associated with inattentional blindness and reduced situational awareness. Findings from past studies and our current work seem reasonable because playing Pokémon Go can be more cognitively demanding compared with other smartphone tasks [15]. This could be because to capture freely roaming Pokémon, phone cameras must be used extensively; moreover, training and battling other Pokémon involve substantial tapping on the touchscreen. While playing Pokémon Go, these activities may impair a pedestrian’s navigational ability when crossing the street, resulting in inattentional blindness and reduced situational awareness. Future research examining the effects of contextual features specific to location-based AR games would be fruitful.

We further found an association of the interaction of unlimited data use and smartphone gaming with inattentional blindness and decreased situational awareness. Chen and Pai [15] also identified this combined effect as a risk factor for risky street-crossing behavior; therefore, attention should be given to smartphone gamers whose Internet data usage is particularly high.

We found that larger smartphone screens (i.e., 5 in. or larger) increased the likelihood of smartphone overuse, inattentional blindness and deafness, and decreased situational awareness. Kim and Sundar [25] found that because large screens facilitate both hedonic and utilitarian uses of smartphones, they were more likely than smaller screens to entice people to adopt smartphones. We speculate that users of phones with larger screens probably have large mobile data allowances and are therefore overusers of smartphones and more likely to suffer from inattentional blindness and decreased situational awareness than are users with small screens. This speculation should be confirmed by future studies that analyze additional data on screen size, usage patterns, and behavior.

Studies (e.g., [26]) have suggested that smartphone addiction among students is associated with depression, anxiety, and sleep problems. Our study demonstrated that smartphone gaming among student pedestrians was associated with smartphone overuse, inattentional blindness and deafness, and lower situational awareness. Efforts should be made to target and educate student smartphone gamers.

One major research limitation of our study arises from the fact that we both observed participants and later interviewed them. Unfortunately, causal inference was not possible; therefore, we investigated simple associations. Moreover, despite adopting random sampling, not all distracted and undistracted pedestrians were selected, because it was impossible to observe all pedestrians walking on the sidewalk and crossing the street. This was another inevitable research limitation. The third research limitation is that the study was conducted beginning in August 2016, immediately following the unprecedented growth in popularity of Pokémon Go (albeit after a noticeable decline in user base). Undoubtedly, our data are representative only of the peak period, but we argue that if another AR game reaches a similar level of popularity, our data may be extensible to the safety risks from playing.

## Conclusions

In conclusion, among various smartphone tasks we considered, playing games such as Pokémon Go was most associated with inattentional blindness and lower situational awareness, whereas listening to music was most associated with smartphone overuse and inattentional deafness. Therefore, playing smartphone games, especially AR games such as Pokémon Go, should not be permitted when crossing the street.

## Abbreviations

4G: Fourth generation
app: Application
AR: Augmented-reality
CI: Confidence interval
dBA: Decibel A weighting
HD: High definition
OR: Odds ratio
US: United States
